# Oncolytic virotherapy with an armed vaccinia virus in an orthotopic model of renal carcinoma is associated with modification of the tumor microenvironment

**DOI:** 10.1080/2162402X.2015.1080414

**Published:** 2015-10-06

**Authors:** Laetitia Fend, Christelle Remy-Ziller, Johann Foloppe, Juliette Kempf, Sandrine Cochin, Luc Barraud, Nathalie Accart, Philippe Erbs, Sylvie Fournel, Xavier Préville

**Affiliations:** a Transgene S.A., 400 boulevard Gonthier d'Andernach, Parc d'innovation, CS80166, Illkirch-Graffenstaden Cedex, France and Institut Gustave Roussy, Unité INSERM 1015 114 rue Edouard-Vaillant, 94805 Villejuif Cedex, France; b Novartis Institutes for Biomedical Research, Analytical Sciences and Imaging, WSJ386, Basel, Switzerland; c Laboratoire de Conception et Application de Molécules Bioactives, Equipe de Biovectorologie, UMR 7199 CNRS-Université de Strasbourg, Faculté de Pharmacie, 74 Route du Rhin- BP60024, Illkirch-Graffenstaden Cedex, France

**Keywords:** Oncolytic virotherapy, renal carcinoma, regulatory T cells, suicide gene therapy, tumor microenvironment, vaccinia virus

## Abstract

Oncolytic virotherapy is an emergent promising therapeutic approach for the treatment of cancer. We have constructed a vaccinia virus (WR strain) deleted for thymidine kinase (TK) and ribonucleotide reductase (RR) genes that expressed the fusion suicide gene *FCU1* derived from the yeast cytosine deaminase and uracil phosphoribosyltransferase genes. We evaluated this construct (VV-FCU1) in the orthotopic model of renal carcinoma (RenCa). Systemic administration of VV-FCU1 resulted in orthotopic tumor growth inhibition, despite temporary expression of viral proteins. VV-FCU1 treatment was associated with an infiltration of tumors by CD8^+^ T lymphocytes and a decrease in the proportion of infiltrating Tregs, thus modifying the ratio of CD8^+^/CD4^+^ Treg in favor of CD8^+^cytotoxic T cells. We demonstrated that VV-FCU1 treatment prolonged survival of animals implanted with RenCa cells in kidney. Depletion of CD8^+^ T cells abolished the therapeutic effect of VV-FCU1 while depletion of CD4^+^ T cells enhanced its protective activity. Administration of the prodrug 5-fluorocytosine (5-FC) resulted in a sustained control of tumor growth but did not extend survival. This study shows the importance of CD4^+^ and CD8^+^ T cells in vaccinia virus-mediated oncolytic virotherapy and suggests that this approach may be evaluated for the treatment of human renal cell carcinoma.

## Abbreviations

5-FC: 5-Fluorocytosine
5-FU: 5-Fluorouracil
APC: AlloPhycoCyanin
BALB/c: Bagg Albino inbred mouse strain
CEE: European Economic Community
CEF: Chicken Embryo Fibroblasts
Cy3: 5.5, 7, Cyanin 3, 5.5, 7
DAB: 3,3′-diaminobenzidine
DAPI: 4′,6-Diamidino-2-Phenylindole
FCU1: chimeric gene encoding yeast cytosine deaminase and uracil phosphoribosyltransferase activities
FITC: Fluorescein isothiocyanate
GFP: Green Fluorescent Protein
GPT: Xanthine/Guanine Phosphoribosyl Transferase
HRP: Horse Radish Peroxidase
IHC: Immuno HistoChemistry
MDSC: Myeloid Derived Suppressor Cells
MOI: Multiplicity Of Infection
PCR: Polymerase Chain Reaction
PerCP: Peridinin Chlorophyll Protein
RenCa: Renal Carcinoma
RPMI: Roswell Park Memorial Institute
RR: Ribonucleotide Reductase
SEM: Standard Error of the Mean
TK: Thymidine Kinase
VV: Vaccinia Virus
WR: Western Reserve

## Introduction

Oncolytic virotherapy is a promising therapeutic option based on the ability of replication competent viruses to replicate and spread within tumors while exerting an oncolytic activity through a direct cytopathic effect. Various oncolytic platforms are being evaluated in clinical studies for their *in vivo* efficacy and first-in-class US approval is expected soon.^1^ Vaccinia viruses (VV) are part of this emerging technology because of their ability to efficiently replicate, lyse host cell and spread across a broad mammalian host range.^2^ We constructed a TK gene-deleted VV and showed that it preferentially replicated in tumors when injected intravenously in mice.^3^ Deletion of the TK gene inhibits viral replication in normal, non-dividing cells, whereas cancer cells have an increased pool of functional nucleotides allowing vaccinia virus replication in the absence of viral TK. This VVTK^−^ was deleted for the viral gene I4L to knock down viral RR. Finally, to further enhance the oncolytic activity of this candidate, the VVTK^−^RR^−^ backbone was armed with the fusion suicide gene named *FCU1* comprising the yeast cytosine deaminase and uracil phosphoribosyl transferase genes.^4^ The resulting chimeric enzyme that is produced by infected cells converts the relatively nontoxic anti-fungal agent 5-FC to 5-Fluorouracil (5-FU), a thymidylate synthase inhibitor which is used to treat several type of cancers. In a previous study, we have demonstrated vector targeting of tumors growing subcutaneously following systemic administration of VVTK^−^ virus armed with this FCU1 fusion gene. More importantly, we also demonstrated that the systemic injection of this construct followed by treatment with 5-FC *per os*, lead to substantial tumor growth delay in nude mice bearing orthotopic liver metastasis of a human colon cancer.^5^

To confirm its genuine promise and reach its full potential in the treatment of cancer, oncolytic virotherapy has to overcome two limitations: first, the delivery of the virus to the tumor and second, the spread of the virus infection through the tumor. Most current preclinical models are inadequate to address these issues because they are based on cells in culture and human xenograft models and lack the confrontation to a functional immune system.^1^ Hence, an orthotopic cancer model in an immunocompetent animal would be a useful tool to understand how to overcome the present constraints of this technology. We evaluated the therapeutic potential of a Western Reserve (WR) strain of our armed VV (VV-FCU1) in an orthotopic model of metastatic RenCa in which RenCa cells injected in one kidney of BALB/c mice will spontaneously metastasize to the lungs.^6,7^ Renal cell carcinoma account for approximately 3% of adult cancers making it the 10th most common malignancy in developed countries.^8^ Characteristics of this pathology include lack of early warning signs and resistance to radio- and chemotherapy. Consequently, (*i*) at the time of diagnosis, one-third of patients display distant metastasis and relapse with metastatic disease occurs in 40% of patients that have undergone nephrectomy; (*ii*) those patients face a poor prognosis while having limited therapeutic options.^9^

We report here that multiple systemic administration of VV-FCU1 via the intra-peritoneal (i.p.) route, leads to control of RenCa tumor growth. This oncolytic activity is associated with a transient detection of viral and chimeric FCU1 protein in kidney tumors. We also noted a modification of the tumor microenvironment caused by an increased infiltration of CD3^+^ T lymphocytes (mainly CD8^+^ T lymphocytes) and a decreased proportion of CD4^+^ Tregs in tumor bearing kidneys as a result of VV-FCU1 oncolytic virotherapy. In the essential presence of CD8^+^ T cells, VV-FCU1 treatment resulted in a significant survival increase of tumor bearing animals. Depletion of CD4^+^ T cells enhanced the therapeutic activity of VV-FCU1. Activation of the suicide gene *FCU1* component by oral gavage with 5-FC did not further enhance survival of the animals but prolonged the control of *in situ* tumor growth.

## Results

### *In vitro* activity of oncolytic vaccinia virus on RenCa and metastatic RenCa cells

**Figure 1.**
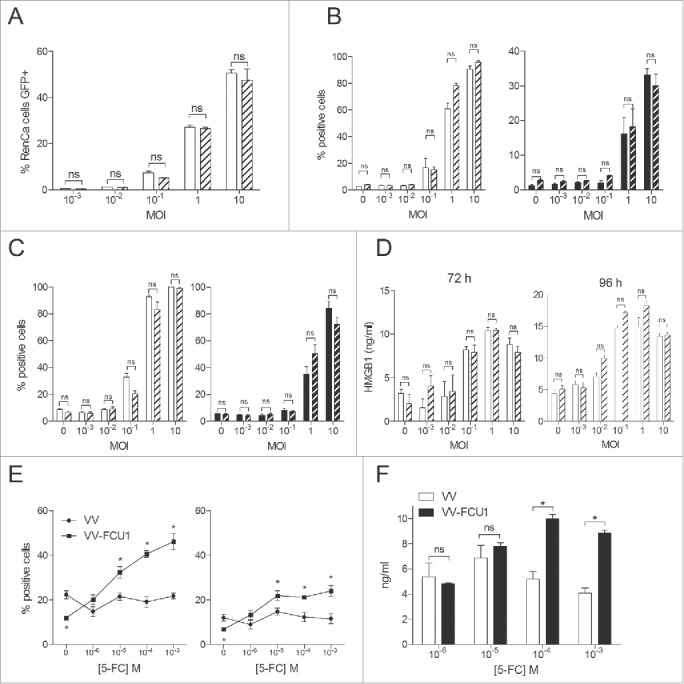
*In vitro* activity of oncolytic vaccinia virus on RenCa and metastatic RenCa cells. (A) *In vitro* infection of RenCa cells (plain histograms) and metastatic RenCa cells (dashed histograms) by VV-GFP (WR VVTK-RR- expressing Green Fluorescent Protein). Cells were infected overnight at the indicated MOI with VV-GFP. The percentage of cells expressing GFP was determined by flow cytometry. (B) *In vitro* cytotoxicity assay. VV-FCU1 was added, at the indicated MOI, over 5 × 10^4^ RenCa (plain histograms) or metastatic RenCa cells (dashed histograms) and incubated for 3 d or (C) 4 d. Percentages of Annexin V (left panel) and Annexin V + Propidium Iodide (right panel) positive cells were determined by flow cytometry analysis. (D) Quantification of HMGB1 by ELISA. Supernatants from RenCa and metastatic RenCa cells infected as described in (B) were collected after 72 and 96 h of incubation and HMGB1 was quantified by ELISA. (E) Effect of 5-FC on VV-FCU1-induced cytotoxicity. RenCa cells (5 × 10^4^) were infected with empty VV or VV-FCU1 vectors (MOI 10^−2^) for 4 d in presence of the indicated concentrations of 5-FC. Percentages of Annexin V (left panel) and Annexin V + Propidium Iodide (right panel) positive cells were determined by flow cytometry. (F) Quantification by ELISA of HMGB1 in the supernatants of RenCa cells incubated with 5-FC following transduction with VV or VV-FCU1. Each bar or data point represent the mean ± SEM of three independent experiments. (ns: not significant, * *p* < 0.05).

To verify the ability of the WR strain of VV to infect RenCa and metastatic RenCa cells, those cells were infected overnight at the indicated multiplicity of infection (MOI) with a VV deleted for TK and RR expressing GFP instead of FCU1. We observed a dose dependent and equivalent infection of both type of cells by VV-GFP (Fig. 1A). To test the oncolytic activity of VV-FCU1, RenCa, and metastatic RenCa cells were infected at the indicated MOIs for a maximum of 4 d. Three days later, we observed an increased percentage of early apoptotic RenCa and metastatic RenCa cells at MOI 10^−1^ and above of VV-FCU1, as determined by Annexin V staining (Fig. 1B, left panel). One extra day of infection resulted in slightly increased percentages of early apoptotic RenCa and metastatic RenCa cells (Fig. 1C, left panel). An increase in the proportion of necrotic or late apoptotic RenCa and metastatic RenCa cells, as determined by Annexin V positive cells incorporating propidium iodide, was observed only at MOI 1 and above, both after 3 d and 4 d of incubation (Fig. 1B and C right panels). To investigate whether RenCa cell death induced by VV-FCU1 could be classified as immunogenic,^10-12^ we measured HMGB1 and ATP release. The highest MOIs of VV-FCU1 (10^−1^, 1, and 10, Fig. 1D) were associated with an increase of HMGB1 release that was detectable at 72 h and 96 h. There was no difference in HMGB1 release between RenCa and metastatic RenCa cells. In such conditions, we could not detect ATP release in supernatants of both cell types (data not shown). To test the functionality of the FCU1 strategy, RenCa cells were incubated for 4 d with mock VV or VV-FCU1 at a non-oncolytic MOI (10^−2^) while increasing concentrations of 5-FC were added to the culture medium at day 2 in order to increase the antitumoral activity of VV-FCU1. A significant increase in the proportion of apoptotic or necrotic RenCa cells was noted as concentrations of 5-FC increased in the group treated with VV-FCU1 (Fig. 1E). This results from the cytotoxic and bystander effects of 5-FU produced by the FCU1 chimeric enzyme.^5^ The highest 5-FC concentrations (10^−4^ and 10^−3^ M) were associated with a modest elevation of HMGB1 release (Fig. 1F) in the VV-FCU1-treated group but we could not detect ATP release in these conditions (data not shown).

### *In vivo* tumor growth inhibition by VV-FCU1

**Figure 2.**
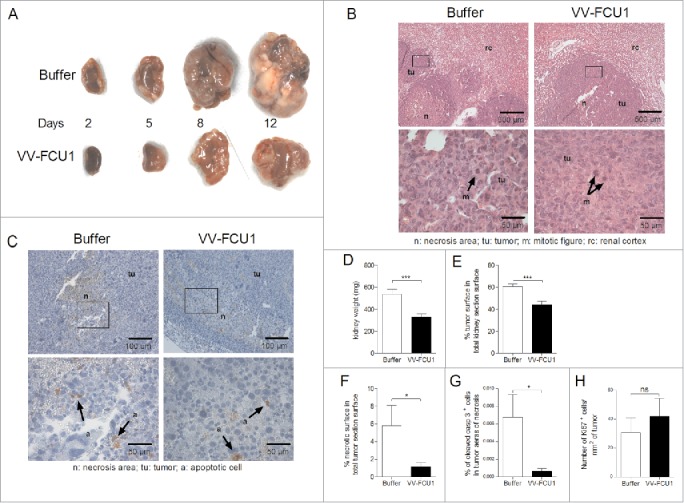
Tumor growth limitation effects of VV-FCU1. RenCa cells were injected into the subcapsular space of the left kidney of female BALB/c mice. Twelve days after cells implantation, mice were treated i.p. with VV-FCU1 or buffer (day 0). Those injections were repeated at day 3 and 6 after the first one. The orthotopic tumors were resected 2, 5, 8, and 12 d after the first VV-FCU1 injection. (A) Representative macroscopic view of the sampled orthotopic tumors is shown in the indicated treatment groups. (B) Representative hematoxylin eosin stained tissue sections of tumors excised at day 8 after the beginning of VV-FCU1 injections, showing renal cortex, tumor and tumor necrotic areas in buffer- and VV-FCU1-treated animals. Upper panels: 40× magnification, lower panels: 400× magnification. Areas magnified in the lower panels are framed in the upper panels. (C) Representative immunodetection of cleaved caspase 3. Sections of tumors excised at day 8 following onset of oncolytic virotherapy, were processed for the detection of cleaved caspase 3 by IHC. Upper panels: 100× magnification, lower panels: 400× magnification. Areas magnified in the lower panels are framed in the upper panels. (D) Weight of tumor bearing kidneys sampled at day 8 after the first injection of VV-FCU1. (E) Percentage of tumor surface in total kidney section surface at day 8 following onset of oncolytic virotherapy. Stained sections as in (B) were scanned with a nanozoomer. Tumor areas were delineated manually using the Calopix software to calculate the percentage of tumor tissue within total kidney section. (F) Percentage of necrotic surface in total kidney tumor section surface at day 8 after the beginning of VV-FCU1 treatment. Stained sections as in (B) were scanned with a nanozoomer. Necrotic areas were delineated manually using the Calopix software to calculate the percentage of necrotic tissue within total kidney tumor section. (G) Percentage of apoptotic cells in total kidney tumor section surface at day 8 after initiation of VV-FCU1 therapy. Sections obtained in (C) were scanned with a nanozoomer and the percentage of cleaved caspase 3 positive cells within necrotic areas was determined using the Calopix software. (H) Percentage of proliferating cells in total kidney tumor section surface at day 8 following onset of oncolytic virotherapy. Sections were processed for the staining of Ki67^+^ cells by IF and were scanned with a nanozoomer. The percentage of Ki67^+^ cells was determined using the Calopix software. Results are from two independent experiments, each including 10 animals. (ns not significant, *p <0.05, ***p < 0.001).

We next studied the oncolytic activity of VV-FCU1 in the orthotopic RenCa tumor model. Oncolytic virotherapy (3 injections at a 3-d interval) started 12 d after injection of RenCa cells in the renal capsule of BALB/c mice. At the indicated times, mice were euthanized and their tumor bearing kidneys were collected for macroscopic and histological characterization. We observed an inhibition of tumor growth following VV-FCU1 injection at the time points tested (Fig. 2A). On sections from tissues harvested at day 8 after the start of VV-FCU1 treatment, the proportion of tumor tissue over that of normal kidney tissue as well as the percentage of necrotic surface from total tumor surface were quantified from nanozoomer scans of hematoxylin eosin stained tissue sections (Fig. 2B). Similarly, sections from tumor bearing kidneys harvested at day 8 were also used for the immunodetection and quantification of cleaved caspase 3 in necrotic areas of tumors (Fig. 2C). Eight days after the start of treatment, kidneys from VV-FCU1-treated animals were significantly lighter in comparison to buffer-treated animals (Fig. 2D). Consistent with this observation, tumor invasion was significantly limited (Fig. 2E) and the percentage of necrotic area from tumor tissue was less elevated (Fig. 2F) in VV-FCU1 treated animals. We also observed that necrotic areas from tumor tissue contained less apoptotic cells after treatment by VV-FCU1 (Fig. 2G), suggesting that these necrotic areas contained only dead cells. To determine whether difference in tumor growth upon VV-FCU1 treatment could originate from a difference in cell proliferation, tissue sections harvested at day 8 after onset of oncolytic virotherapy were also analyzed for Ki67 expression. We could not detect a significant difference between buffer and VV-FCU1-exposed tissue (Fig. 2H).

### Detection of viral proteins *in vivo* and monitoring of antiviral adaptive response

**Figure 3.**
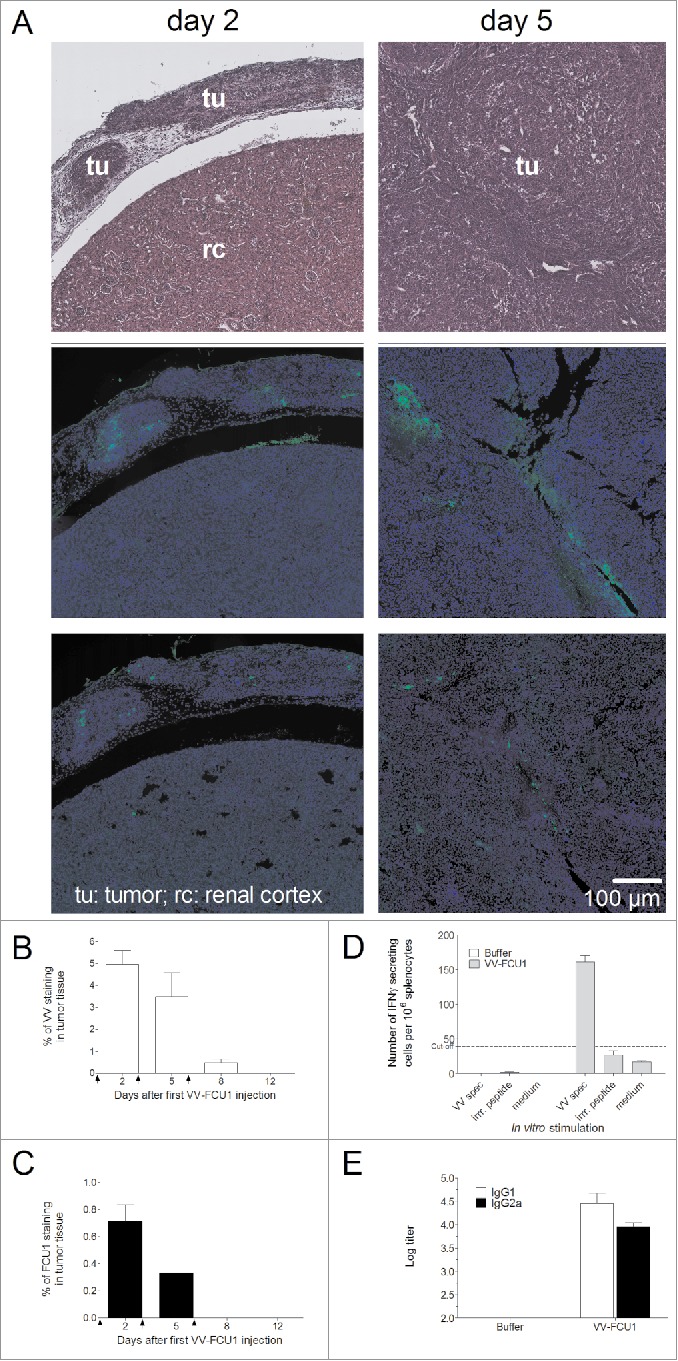
Transient expression of viral proteins *in vivo* and evaluation of adaptive cellular and humoral antiviral responses. (A) RenCa cells were injected into the subcapsular space of the left kidney of female BALB/c mice. Twelve days after cells implantation, mice were treated i.p. with VV-FCU1 or buffer (day 0). Those injections were repeated at day 3 and 6 after the first one. The orthotopic tumors were resected 2 and 5 d after the first VV-FCU1 injection and analyzed by H&E staining (top panels) to decipher between normal and tumor tissue as indicated, and by IF on adjacent sections with a polyclonal rabbit anti-vaccinia as the primary antibody (middle panels) or a polyclonal rabbit anti-FCU1 as the primary antibody (bottom panels). Magnification: ×5. (B) Detection of VV protein expression by IF. Percentages of VV stained areas were measured on the entire tumor section from nanozoomer acquired images using the Calopix software. (C) Detection of FCU1 protein expression by IF. Percentages of FCU1 stained areas were measured on the entire tumor section from nanozoomer-acquired images using the Calopix software. Arrows on graphs represent the days of VV-FCU1 injection. (D) Evaluation of adaptive antiviral cellular response. Three days after i.p. injection of buffer or VV-FCU1, splenocytes from treated mice were used to detect cellular immunity to VV backbone by IFNγ ELISpot assay upon *in vitro* stimulation with an H-2D^d^ restricted VV-specific peptide. The specificity of the cellular immune response was controlled by an irrelevant peptide. (E) Evaluation of adaptive antiviral humoral response. Six days after i.p. injection of buffer or VV-FCU1, sera from buffer or VV-FCU1 treated mice were used to detect humoral immunity to VV backbone by ELISA. Results are from two independent experiments, each including five animals.

To better understand the *in vivo* oncolytic effect of VV-FCU1, we monitored the expression of vaccinia virus protein and FCU1 in tissue sections of tumor bearing kidneys. Oncolytic virotherapy (3 injections at a 3-d interval) started 12 d after injection of RenCa cells in the renal capsule of BALB/c mice. Both viral proteins and chimeric FCU1 protein could be detected in tumor tissue 2 d following 1 or 2 injections of virus. Under the same conditions, we could not detect expression of neither viral proteins nor FCU1 protein in normal tissue adjacent to tumor (Fig. 3A). Expression of viral proteins was transient and disappeared despite repeated injection of VV-FCU1 as determined by quantification of the percentage of VV staining in tumor tissue from nanozoomer-acquired tissue section (Fig. 3B). A similar observation was made for the chimeric FCU1 protein that was no longer detected 2 d after the third injection of VV-FCU1 (Fig. 3C). We postulated that the adaptive immune response against vaccinia virus could be involved in this transient expression of viral antigens. We therefore monitored the early adaptive antiviral response to VV backbone. A specific systemic anti VV cellular response could be detected by ELISpot as early as 3 d after i.p. injection with 10^7^ plaque-forming unit (pfu) of VV-FCU1 (Fig. 3D). Regarding the adaptive humoral response, important IgG1 and IgG2a titers to VV-FCU1 could be detected by ELISA as early as 6 d after VV-FCU1 injection (Fig. 3E).

### Characterization of immune infiltrates in early orthotopic RenCa tumors

**Figure 4.**
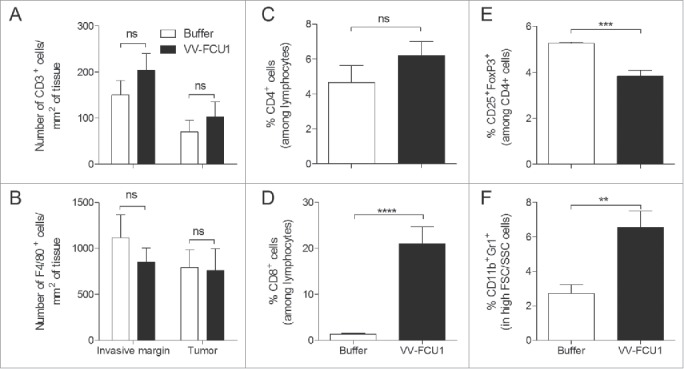
Characterization of tumor-infiltrating immune cells. (**A**) RenCa cells were injected into the subcapsular space of the left kidney of female BALB/c mice. Twelve days after cells implantation, mice were treated i.p. with VV-FCU1 or buffer (day 0). Those injections were repeated at day 3 and 6 after the first one. The orthotopic tumors were resected 8 d after the first VV-FCU1 injection and analyzed by IF with a polyclonal rabbit anti mouse CD3 or a rat anti mouse F4/80 primary as primary antibodies. Numbers of CD3^+^ cells per mm^2^ at the invasive margin or within tumor tissue were calculated on the entire tumor section from nanozoomer-acquired images using the Calopix software. (B) Numbers of F4/80^+^ cells per mm^2^ at the invasive margin or within tumor tissue were calculated on the entire tumor section from nanozoomer-acquired images using the Calopix software. (C) In a different set of experiments, tumors resected 8 d after the first VV-FCU1 injection were crushed and processed for determination by flow cytometry of the percentage of CD4^+^ cells among lymphocytes; (D) the percentage of CD8^+^ cells among lymphocytes; (E) the percentage of Treg cells among CD4^+^ cells; and (F) the percentage of MDSC among cells with high FSC and SSC values. Results are from two independent experiments, each including five animals. (ns: not significant, **p < 0.01, ***p < 0.001, ****p < 0.0001).

As immune cells such as CD3^+^ tumor infiltrating T lymphocytes and F4/80^+^ macrophages may have positive or negative roles in the control of tumor growth,^13,14^ the proportion of infiltrating CD3^+^ and F4/80^+^ cells was studied by immunofluorescence (IF) on tumor tissue section harvested at day 8 after onset of oncolytic virotherapy. In comparison to animals injected with buffer, the number of CD3^+^ cells per square millimeter of both the invasive margin and tumor tissue tended to be higher in VV-FCU1 treated animals (Fig. 4A). Tumor invasive margins from VV-FCU1 treated animals tended to have a lower density of F4/80^+^ cells than those of buffer-treated animals, whereas tumors from buffer- and VV-FCU1-treated animals appeared to have similar densities of F4/80^+^ cells (Fig. 4B). In parallel, we conducted immunophenotyping studies on lysates from other tumors harvested at day 8 after the onset of VV-FCU1 treatment. In comparison to those from buffer-treated animals, lysates from tumors from VV-FCU1-treated animals were characterized by a slightly higher proportion of CD4^+^ cells within cells displaying size and scatter properties of lymphocytes (Fig. 4C). In contrast, the proportion of CD8^+^ cells within the lymphocytes area of the side/scatter dot plot was significantly higher in the VV-FCU1 treated group (Fig. 4D). The proportion of CD4^+^ cells displaying regulatory T cells characteristics, as evaluated by the expression of CD25 and FoxP3, was significantly lower in the tumor lysates from VV-FCU1-treated animals in comparison to that of controls (Fig. 4E). We also measured the proportion of CD11b^+^Gr1^+^ cells in the same tumor lysates within cells displaying high side and scatter properties. CD11b^+^ and Gr1^+^ characterize myeloid derived suppressor cells (MDSC),^15^ but monocytes and granulocytes can also be found within CD11b^+^Gr1^+^ cells. We observed a higher proportion of CD11b^+^Gr1^+^ cells within tumor lysates from VV-FCU1-treated animals in comparison to that of controls (Fig. 4F).

### Therapeutic effect of VV-FCU1 in ectopic and orthotopic RenCa models, impact of CD8^+^ and CD4^+^ cells

**Figure 5.**
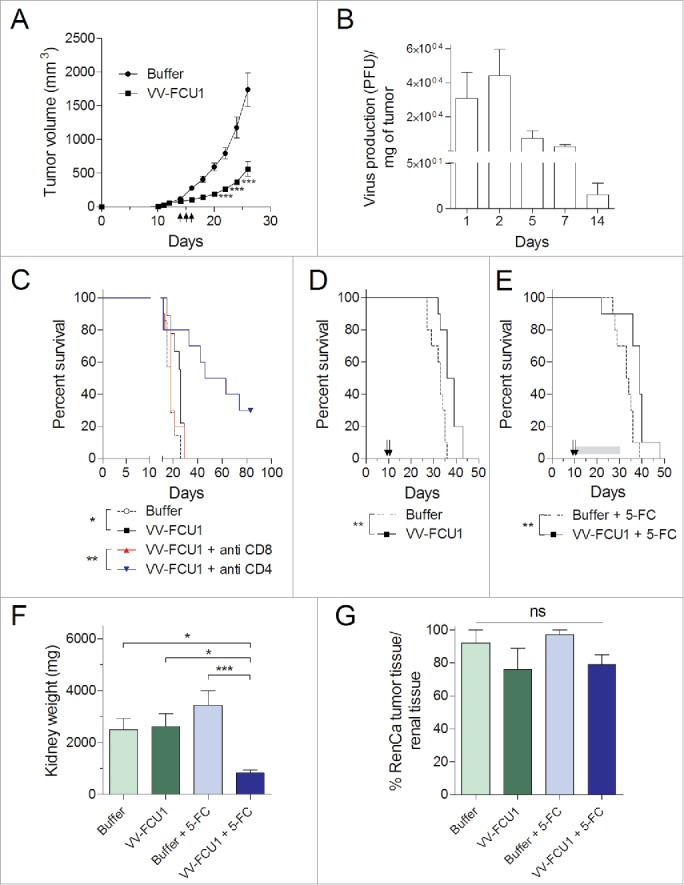
Therapeutic effect and replication of VV-FCU1 *in vivo*. (A) RenCa cells (3.10^5^) were injected subcutaneously in the right flank of female BALB/c mice. When tumor sizes reached 100 mm^3^, mice received three intratumoral (i.t.) injections of 10^7^ pfu of VV-FCU1 at 3-d intervals (symbolized by black arrows). Tumor growth was monitored with a caliper and is reported. (B) *In vivo* replication of VV-FCU1 in RenCa s.c. tumors. One hundred cubic millimeter RenCa tumors were injected with 10^7^ pfu of VV-FCU1. At indicated time points, tumors were sampled, crushed, and sonicated for titration of remaining virus on CEF. (C) RenCa cells (3.10^5^) were injected subcutaneously in the right flank of female BALB/c mice (day 0). Specific antibodies were used at day 3 and day 4 to deplete CD8^+^ and CD4^+^ cells. Buffer or VV-FCU1 (10^7^ pfu) injections were done i.t. at day 11, day 14, and day 15. Percentages of mice survival are shown. (D) RenCa cells (10^4^) were injected into the subcapsular space of the left kidney of female BALB/c mice. Eight days after cells implantation, mice were treated i.p. twice with a 3-d interval between injections as symbolized by black arrows. Percentages of mice survival are shown. (E) Same as in (D) but 5-FC (200 mg/kg/day) was given to the animals *per os* for 21 d as symbolized by the gray bar. (F) Orthotopic tumor weight. In a different set of experiments, at the end of the 5-FC treatment that is 32 d after RenCa cells implantation, tumorous kidneys were resected and their weights were recorded. (G) Analysis of orthotopic tumor invasion of kidney. Explanted tumorous kidneys were processed for Hematoxylin/Eosin (HE) staining. From nanozoomer-acquired images of HE-stained tissue sections, the percentage of tumor surface among the entire kidneys was calculated. Results are from two independent experiments, each including three to five animals. (ns: not significant, *p < 0.05, **p <0.01, ***p < 0.001).

The *in vivo* oncolytic activity of VV-FCU1 toward s.c. implanted RenCa tumors was investigated in BALB/c mice. Treatment with three intra tumoral injections of VV-FCU1 significantly inhibited the growth of subcutaneously-established RenCa tumors (Fig. 5A). Replication of VV-FCU1 was investigated *in vivo* after a single injection of 10^7^ pfu in subcutaneous RenCa tumors. As shown in Fig. 5B, there was no replication of VV-FCU1 within RenCa tumors and the virus disappeared with time, thus confirming the transient feature of VV-FCU1 treatment in immunocompetent BALB/c mice. We next investigated the role of CD8^+^ and CD4^+^ lymphocytes in the therapeutic activity of VV-FCU1. Antibody-mediated depletion of CD8^+^ cells prior VV-FCU1 treatment abolished the therapeutic effect of VV-FCU1. In contrast, CD4^+^ cell depletion enhanced VV-FCU1 induced protection (Fig. 5C).

In the orthotopic model of renal cell carcinoma, when VV-FCU1-based therapy was given 12 d after tumor implantation, we could not detect any benefit in terms of survival for the animals treated by VV-FCU1 (data not shown), despite the described *in situ* tumor growth limitation effects (Fig. 2). Therefore, we started to treat the animals 8 d following tumor grafting and compared the efficacy of two versus three therapeutic injections based on the rationale that the FCU1 chimeric protein is not detected by IF 2 d after the 3rd injection of VV-FCU1 (Fig. 3C). Under such schedule, we observed that two injections of VV-FCU1 resulted in a significant prolongation of animal survival (*p* = 0.0013) as determined by Log-rank test (Fig. 5D). Addition of 5-FC gavage to VV-FCU1 treatment to induce the *in situ* production of 5-FU, did not further enhance the survival of the animals (*p* = 0.0025, Fig. 5E). Of note, a 3rd injection of VV-FCU1 did not enhance animal survival in this model even when animals were fed with 5-FC (data not shown). At the end of 5-FC treatment, i.e. 32 d after injection of RenCa cells, tumor bearing kidneys were collected and processed, in each group, for macroscopic examination and microscopic characterization. We observed that VV-FCU1 + 5-FC treatments resulted in a sustained control of tumor growth *in situ* as kidneys from this experimental group were significantly lighter as compared to the other groups (Fig. 5F). Nevertheless hematoxylin/eosin staining of tumor tissue section revealed that kidneys from different groups were similarly almost completely invaded by tumor cells (Fig. 5G).

### Characterization of immune cells infiltrates in advanced kidney tumors

**Figure 6.**
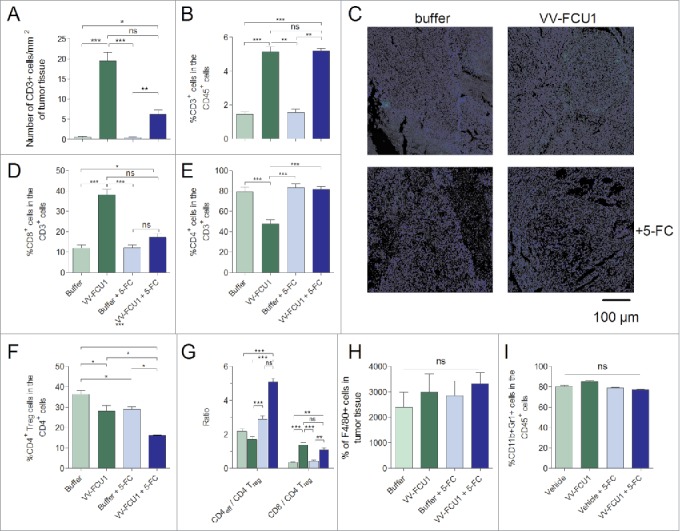
Characterization of the immune infiltrates in kidney tumors. RenCa cells (10^4^) were injected into the subcapsular space of the left kidney of female BALB/c mice. Eight days after cells implantation, mice were treated i.p. twice with a 3-d interval between injections. At the end of the 5-FC treatment, that is 32 d after tumor implantation, tumorous kidneys were resected and processed either for analysis by IF of CD3 and F4/80 markers or by flow cytometry of CD3, CD8^+^, CD4^+^, Treg, and MDSC markers. (A) Numbers of CD3^+^ cells per mm^2^ of tumor tissue were calculated on the entire tumor section from nanozoomer-acquired images using the Calopix software (B) Flow cytometry determination of the percentages of CD3^+^ lymphocytes among CD45^+^ cells. (C) Representative IF images of the results presented in (A) 5× Magnification. (D), (E) Flow cytometry determination of the percentages of CD8^+^ and CD4^+^ lymphocytes among CD3^+^ lymphocytes, respectively. (F) Flow cytometry determination of the percentages of CD4^+^ Treg lymphocytes among CD4^+^ lymphocytes. (G) Flow cytometry determination of ratios of conventional CD4^+^ and CD8^+^ lymphocytes over CD4^+^ Treg lymphocytes. (H) Numbers of F4/80^+^ cells per mm^2^ of tumor tissue were calculated on the entire tumor section from nanozoomer-acquired images using the Calopix software. (I) Flow cytometry determination of the percentages of CD11b+Gr1+ cells among CD45^+^ cells. For each graph, mean ± SEM are represented. Results are from two independent experiments, each including five animals. (ns not significant, *p < 0.05, **p < 0.01, ***p < 0.001).

We characterized by IF and flow cytometry immunophenotyping the immune infiltrates of those late stage collected tumors. We observed by IF analysis that kidneys from VV-FCU1-treated animals contained a higher number of CD3^+^ lymphocytes per square millimeter of tumor tissue in comparison to buffer-treated animals (Fig. 6A, C). From these IF analyses, tumors from VV-FCU1 + 5-FC treated animals appeared to contain a lower density of CD3^+^ cells/mm^2^ in comparison to tumors from VV-FCU1 treated animals. Flow cytometry immunophenotyping indicated that CD45^+^ immune infiltrated cells contained a significantly higher percentage of CD3^+^ lymphocytes upon VV-FCU1 treatment of the animals whatever the addition of 5-FC or not (Fig. 6C). The proportion of CD8^+^ cells among CD3^+^ lymphocytes was significantly higher in VV-FCU1-treated animals in comparison to respective controls (Fig. 6D). Of note, the proportion of CD8^+^ infiltrating the tumor appeared to be higher in VV-FCU1-treated animals in comparison to those treated with VV-FCU1 and fed with 5-FC. Conversely, the proportion of CD4^+^ cells among CD3^+^ lymphocytes was significantly lower in VV-FCU1-treated animals in comparison to all other groups (Fig. 6E). The proportion of CD4^+^ Treg cells was lower in VV-FCU1-treated groups compared to respective controls (Fig. 6F) and the proportion of Treg cells within CD4^+^ lymphocytes was significantly reduced after VV-FCU1 + 5-FC treatment in comparison to VV-FCU1 treatment alone. Consequently, we observed that the ratio of conventional CD4^+^ effector cells to CD4^+^ Treg lymphocytes was the highest in the group treated with VV-FCU1 + 5-FC, and that of CD8^+^ effector cells to CD4^+^ Treg lymphocytes was higher in groups that received VV-FCU1 in comparison to their respective controls (Fig. 6G).

The density of F4/80^+^ cells was also analyzed from nanozoomer scans of tumor sections sampled at day 32. We did not observe any significant differences among the groups for this parameter (Fig. 6H). Flow cytometry immunophenotyping of CD45^+^ cells exhibiting CD11b^+^ and Gr1^+^ markers indicated also no differences for this parameter among the four groups of our experiment (Fig. 6I).

## Discussion

Oncolytic virotherapy is a promising treatment modality for the therapeutic armamentum to fight cancer. The number of current and recently completed clinical trials based on oncolytic viruses reflects this interesting therapeutic potential.^1^ However, clinical trial results often do not fully meet expectations. In this respect, more predictive and robust preclinical models could be helpful. Most importantly, as viruses are entities that strongly stimulate both the innate and adaptive arms of the immune system, preclinical modeling on a fully immune-competent background is of utmost importance to better understand their *in vivo* mechanism of action in the context of a tumor-associated inflammatory environment.

We have constructed a vector to increase the selective delivery of a suicide gene to tumor cells and evaluated *in vitro* its ability to infect and kill RenCa cells.^5^ The oncolytic activity of VV-FCU1 toward those mouse tumor cells was rather poor compared to that displayed against numerous human tumor cell lines (data not shown). At the highest MOI, we observed features of apoptotic death on remaining living cells. Of note, the infection capacity and the oncolytic activity over RenCa cells and a metastatic clone of RenCa cells was equivalent. In our hands, the release of HMGB1 by RenCa and metastatic RenCa cells treated with VV-FCU1 was comparable to that of anthracycline based chemotherapies.^16^ This observation contrasts results from others and indicating that VV-FCU1 is not a strong inducer of apoptotic immunogenic cell death from mouse tumor cell lines.^17^ We demonstrated *in vitro* the functionality of the suicide gene strategy as addition of increasing quantities of the pro-drug 5-FC in the culture medium, to a non-toxic dose of VV-FCU1, resulted in an increased cytotoxicity toward RenCa cells. Local production of 5-FU did not increase the level of HMGB1 release thus confirming that 5-FU is not an inducer of immunogenic cell death.^18^

We observed a control of orthotopic tumor growth for VV-FCU1-treated animals in comparison to buffer-treated mice, up to late stages (32 d) of tumor evolution provided that animals were given 5-FC to activate local production of 5-FU. In early stages (up to 12 d), this tumor growth control was associated with a reduction of necrotic areas and numbers of apoptotic cells within these necrotic areas. As shown by detection of Ki67, VV-FCU1 treatment did not alter the proliferating capacity of RenCa cells, thus suggesting that control of tumor growth by VV-FCU1 is due to its oncolytic activity and not to a cytostatic activity.

VV-FCU1 demonstrated its ability to target tumor tissue within a kidney following systemic injection, as the expression of VV-related and FCU1 proteins was restricted to tumor areas within sampled kidneys. Detection of VV-FCU1 was however transient and it is likely that the conjunction of the lack of *in vivo* replication in murine RenCa tumors and the rapid cellular and humoral responses detected in mice toward VV backbone account for this observation.

VV-FCU1 treatment was associated with long lasting (up to day 32) increased infiltration of tumors by immune cells among which CD3^+^CD8^+^ T lymphocytes appear to be essential for the *in vivo* control of RenCa tumor growth. This indicates that the therapeutic effect of VV-FCU1 in immunocompetent animal is not solely dependent on the oncolytic properties of the virus and that effector CD8^+^ T cells are required. In contrast, depletion of CD4^+^ cells dramatically enhanced the effect of VV-FCU1-mediated oncolytic virotherapy. This observation is at first contrasting the absolute requirement of CD8^+^ T cells for an efficient oncolytic activity as CD4^+^ T cells are in general required for the maturation of CD8^+^ cytotoxic T lymphocytes. However, it has been shown that CD4^+^ T cell help is not absolutely required for the induction of an efficient CTL response.^19,20^ Alternatively, depletion of CD4^+^ T cells could decrease tumor infiltrating CD4^+^ Treg cells and thus foster the activity of tumor infiltrating CD8^+^ T cells.

Mice implanted with orthotopic RenCa tumors had a significant longer survival following treatment with VV-FCU1 in comparison to controls. However, oral gavage with 5-FC to locally produce 5-FU and increase cytotoxicity within tumors did not further enhance survival time as it would have been expected from the *in situ* tumor growth inhibitory effect seen on tumor-bearing kidneys at day 32. This suggests that turning on the suicide gene strategy has induced a higher antitumor activity in the early stages, thus a better control of tumor growth, and that this gap has remained until the day of our investigation, while tumor was completely invading normal tissue. This would explain why kidneys sampled at day 32 are equally almost completely invaded by tumor tissue, despite the marked control of tumor growth in the VV-FCU1 + 5-FC group.

The metastatic nature of the RenCa orthotopic model could explain this lack of associated therapeutic benefit and future work should aim at evaluating *in vivo* the effect of VV-FCU1 on RenCa metastasis. Indeed, either metastatic niche may be not accessible to VV-FCU1 or CD8^+^ T cells may be useless to control the growth of metastatic RenCa cells as it has been shown that NK cells controlled RenCa spontaneous metastasis and influenced survival.^21^

Recent results from a clinical trial in hepatocellular carcinoma positively emphasized the induction of anticancer immunity as a result of VV-mediated oncolytic virotherapy.^2^ In this respect, we observed a pronounced and sustained (up to day 32) infiltration of tumors with CD3^+^CD8^+^ positive cells in VV-FCU1-treated animals in comparison to controls. As data is accumulating and is being currently validated in clinical trials that a T cell-infiltrated and -inflamed tumor microenvironment may be a relevant predictive biomarker for clinical benefit, induction of CD3^+^CD8^+^ T cell infiltration in tumors by VV-FCU1 is of utmost importance.^22,23^ The mechanism underlying this observation is not known, but it is likely that CXCR3 and its ligands CXCL9 and CXCL10 are involved as it was recently demonstrated in an epicutaneous model of VV infection.^24^

In conclusion, although oncolytic vaccinia virus is not adapted to the infection of murine cells, VV-FCU1-mediated oncolytic virotherapy demonstrated tumor targeting associated with a therapeutic effect in both ectopic and orthotopic model of RenCa. Most importantly, VV-FCU1 treatment modified the CD8^+^/CD4^+^ Treg ratio in the tumor microenvironment. This study constitutes a basis for improvement of this therapeutic approach and a start point for evaluation of oncolytic virotherapy in combination with experimental or currently approved therapeutics for metastatic renal cell carcinoma in the neo-adjuvant, adjuvant and metastatic settings.

## Material and methods

### Animals

Six-week old BALB/c mice (Charles River Laboratories) were housed in a specific-pathogen free animal facility and acclimated for one week before the experiments. All *in vivo* experiments were performed in full compliance with the CEE directive 2010/63 of 22 September 2010 relating to the protection of animals used for experimental or other scientific purposes and in compliance with the French law, décret n° 2013–118 of 1st February 2013.

### Cell line

The murine renal cell carcinoma cell line (RenCa) was provided by the American Type Culture Collection. The RenCa cells were maintained in RPMI-1640 medium supplemented with 10% fetal bovine serum. The cells were cultured at 37°C in a 5% CO_2_ atmosphere and routinely passaged by trypsin treatment in 175 cm^2^ flasks. A metastatic RenCa cell line was generated from lung metastases of BALB/c mice orthotopically implanted with RenCa cells. The metastatic RenCa cells were expanded and cultured similarly to the parental RenCa cell line.

### Oncolytic vaccinia virus

VV-FCU1 is a double deleted TK^−^ and RR^−^ engineered WR strain (WRVVTK^−^RR^−^/FCU1) vaccinia virus expressing FCU1 from the disrupted TK gene. For TK inactivation, successful recombination events were selected as described.^5^ The WRTK shuttle plasmid contains the FCU1 gene under the control of the vaccinia synthetic early/late p11K7.5 promoter surrounded by portions of the vaccinia J2R (TK) gene, which allows homologous recombination into this locus.^25^ Chicken embryo fibroblasts (CEF) were infected with WR wild type, then transfected with the WRTK shuttle plasmid by CaCl_2_ precipitation. The cells were incubated for 48 h at 37°C and then used to infect the TK^−^deficient 143B cells in selection medium containing 5-bromo-2′-deoxyuridine at final concentration, of 150 µg/mL (Sigma). Positive TK^−^ plaques were isolated and selected for a second cycle in 143B cells in presence of 5-bromo-2′-deoxyuridine to obtain a WRTK-/FCU1.

For RR inactivation, we used the WRI4L shuttle plasmid containing the *E. coli* xanthine-guanine phosphoribosyltransferase (GPT) gene surrounded by the flanking sequences of the vaccinia I4L (RR) gene. In this construction, the selection marker GPT is placed between two homologous sequences in the same orientation. Recombination (producing RR inactivation) was performed into WRTK^−^/FCU1 virus using the WRI4L plasmid expressing the GPT gene. The generation of recombinant virus based on GPT selection was previously described in detail.^25^ Briefly, recombinant WRVVTK^−^RR^−^/FCU1/GPT^+^ viruses were isolated by mycophenolic acid selection and multiple purification steps. The selection marker was easily eliminated by several passages without selection allowing the growth of GPT^−^ recombinant WR obtained after intragenic homologous recombination between the two sequences flanking the GPT gene.

VV-FCU1 structures were confirmed by multiple PCRs. Final recombinant VV-FCU1 virus was amplified in BHK21, purified in sucrose gradient and virus stocks were titrated on CEF by plaque assay.

### *In vitro* infection assays of RenCa cells by VV-FCU1

RenCa and metastatic RenCa cells were infected in suspension by VV-FCU1 or indicated derivatives at a MOI ranging from 10 to 10^−3^. To determine the percentage of infected RenCa and metastatic RenCa cells, a total of 1.10^6^ cells per well were plated in six-well culture dishes in 3 mL of RPMI-1640 medium and infected overnight with our VV backbone expressing the green fluorescent protein (GFP) instead of FCU1. The percentage of GFP^+^ cells was determined by flow cytometry (BD FACS^®^ Canto A). To determine the oncolytic activity of VV-FCU1 over RenCa and metastatic RenCa cells, a total of 5.10^4^ cells per well were plated in six-well culture dishes in 2 mL of RPMI-1640 medium and infected with VV-FCU1 for a time period of 4 d after which, cell death was measured by flow cytometry using Annexin V/propidium iodide staining. To test the cytotoxic effect of 5-FU formation during infection by VV-FCU1, RenCa cells (5.10^4^) were comparatively infected in suspension with empty VV (WR VVTK^−^RR^−^ backbone) and VV-FCU1 at an MOI of 10^−2^ as described above. A concentration range of 5-fluorocytosine (10^−6^ to 10^−3^ M) was added to the cultures 48 h after the beginning of the infection. Viability was after assessed 4 d as described above by flow cytometry. The hallmark of immunogenic cell death, ATP and HMGB1 release, were measured from 72 and/or 96 h culture supernatants according to manufacturers recommended procedures (Promega and IBL, respectively).

### *In vivo* therapeutic activity in mouse RCC model

For the subcutaneous tumor model, 3.10^5^ RenCa cells (in 100 µL PBS) were injected subcutaneously into the right flanks of mice. Mice were treated with VV-FCU1 or buffer when tumors reached the size of approximately 100 mm^3^ as measured by a caliper using the following formula: V = 4/3π × (length/2) × (width/2) × (depth/2). Mice were sacrificed when tumor size reached 2,000 mm^3^. For the orthotopic tumor model, mice were anesthetized with isoflurane. ^26^ A lateral incision was made in the left flank of each mouse. With the help of a 0.3 mL insulin syringe equipped with a 30 gauge needle, 10^4^ RenCa cells (in 30 µL PBS) were injected into the subcapsular space of the left kidney. The incision was then sewn closed with suture thread. Depending on the experiment, mice were administered i.t. or i.p. with 10^7^ (pfu /100 µL) of VV-FCU1 or buffer, at the indicated times after cell implantation. Injections were repeated once or twice as indicated. Depletion of CD8^+^ and CD4^+^ cells were achieved with two i.p. injections of 200 µg of anti CD8^+^ (clone 53.6.72) or anti CD4^+^ (clone GK1.5) antibodies, respectively, on day 3 and 4 post-RenCa cells implantation. Where specified, 5-FC (200 mg/kg/day) was given *per os* except on weekends (given in the drinking water at the concentration of 25 mg/mL) during three weeks. Onset of 5-FC treatment was 2 d after the beginning of the VV-FCU1 or buffer treatment.

The replication of VV-FCU1 in tumors *in vivo* was evaluated by virus titration. A dose of 1 × 10^7^ pfu of the virus was injected intratumorally (i.t.) into RenCa tumors implanted s.c. in BALB/c mice. Mice were sacrificed at days 1, 2, 5, 7, and 14. Tumors were then harvested from mice, weighted, homogenized in PBS, sonicated and titers were determined on CEF by plaque assay. Viral titers were standardized to milligram of tissue.

### Histology

For histological characterization of tumor sections, kidneys were removed and fixed for 48 h in Tris Zinc fixative at 4°C. ^27^ Organs were dehydrated and embedded in paraffin according to standard procedures. Five micrometer-thick serial sections were obtained, dried and stored at room temperature. Paraffin sections were dewaxed and rehydrated in alcohol then PBS. Sections were stained in Harris Hematoxylin (Sigma) followed by Eosin (R.A.L.), then washed in water. Finally, sections were dehydrated and mounted in Eukitt® (Labonord). Percentages of tumor and necrotic surface were obtained by hand delimitation of tumor tissue from the entire tissue section.

### Immunohistochemistry and immunofluorescence

Paraffin sections were dewaxed, rehydrated in 100% ethanol and endogenous peroxidase activity was blocked by 2% H_2_O_2_ in methanol. Rehydration was pursued in 70% ethanol, 30% ethanol, and finally PBS. Non-specific protein interactions were eliminated by 10% goat serum (Sigma) in PBS. Paraffin sections were then incubated for 1.5 h at room temperature with the following primary antibodies, rabbit anti FCU1 (Transgene), rabbit anti Vaccinia virus (Maine biotechnology), rabbit anti Ki67 (Bethyl Laboratories), rabbit anti mouse CD3 (Dako), rabbit IgG (Affinity BioReagents), rat anti mouse F4/80 (Caltag Medsystems) or rat IgG2a (BD Biosciences). Rat primary antibodies were detected by a secondary rabbit anti-rat IgG antibody (Dako). Rabbit primary antibodies were detected using horseradish peroxidase (HRP)-conjugated dextran polymer goat anti-rabbit IgG (Dako) followed by FITC-labeled tyramide or Cy3-labeled tyramide (Perkin Elmer). Tissue sections were counterstained with DAPI (Sigma) and mounted in Mowiol® (Calbiochem-Merck). Staining specificity was verified by absence of reaction with isotype-matched unspecific antibodies. For detection of apoptotic cells, paraffin sections were then incubated for 1.5 h at room temperature with the primary antibodies rabbit anti cleaved caspase 3 (Cell signaling) or rabbit IgG (Affinity BioReagents). Then, HRP-conjugated dextran polymer goat anti-rabbit IgG (Dako) was used and the HRP activity was visualized by DAB Substrate-Chromogen. Tissue sections were counterstained with Harris Hematoxylin (Sigma). Images were acquired with an optical microscope 90i (Nikon) equipped with 40× objective for visible and epifluorescence analyses. Signal was quantified on section scans (Nanozoomer, Hamamatsu) using the Calopix software (TRIBVN). To this aim, images were split into three channels (red or green and blue). The blue channel allowed quantifying the blue pixels defining the total tumor section surface, while the red (or green) channel measured the red (or green) pixels corresponding to the immunostained cell surface. Numbers of positive cells per surface unit were calculated as (total red surface (or green) pixels/mean individual cell surface/blue pixels total surface) ×100.

### ELISpot assay

Mononuclear cells were obtained from splenocytes by density gradient centrifugation on Lympholyte-M (Cedarlane). One million mononuclear cells in triplicate wells were seeded in 96-well MSIP plates (Millipore) coated with anti-IFNγ capture mAb (Mabtech AB). They were cultured in 200 µL RPMI-1640 medium (Sigma) supplemented with 10% FCS, 50 µM β-mercaptoethanol (Sigma), 40 µg/mL gentamycin (Schering-Plough) and 2 mM L-glutamine (Sigma), in the presence of 1 µg/mL of H-2^d^-restricted vaccinia-specific peptide (SPGAAGYDL) or 1 µg/mL of H-2^d^-restricted irrelevant peptide (TPHPARIGL). After 18 h incubation at 37°C, 5% CO_2_, plates were washed and immunospots were revealed using biotinylated anti-IFNγ detection mAb (Mabtech), Extravidin (Sigma) and BCIP/NBT solution (Sigma). Spots were counted using a CTL Immunospot reader.

### ELISA

ELISA plates (NUNC Maxisorp (4–39454)) were adsorbed with 100 µL suspension of VV-FCU1 at a concentration of 10^7^ pfu per well. Adsorption was performed overnight at 4°C. Wells were then rinsed with washing buffer (PBS + 0.1% Tween 20 + EDTA 10 mM) and saturated with Buffer PBS + 3% BSA, 1 h at room temperature. Sera were diluted (range of dilution: 1/100 to 1/204 800) in PBS + 1% BSA. Plates were then incubated at room temperature for 120 min and rinsed three times with washing buffer. Then, 100 µL of either an anti-IgG1-HRP mAb (BD Biosciences) or anti-IgG2a-HRP mAb (BD Biosciences) (1/10 000) was dispensed per well and incubated at room temperature for 60 min. After washing, 100 µL of ABTS revelation solution (Roche) was added per well. Enzymatic reaction lasted 30 min minimum and the absorbance was read at 405 nm with a spectrophotometer (Tecan).

### Flow cytometry analysis

To characterize the infiltration of renal tumors by immune cells, kidneys were removed, crushed using Gentle Macs (Miltenyi) to prepare cell suspensions, and subsequently stained for 30 min at 4°C with the following monoclonal antibodies : PE-Cy7-conjugated anti-CD45 (clone 30-F11), PerCP-Cy5.5-conjugated anti-CD19 (clone 1D3), APC-conjugated anti-GR1 (clone RB6-8C5), FITC-conjugated anti-CD11b (clone M1/70), APC-Cy7-conjugated anti-CD3 (clone 145-2C11), APC-conjugated anti-CD4^+^ (clone RM4-5), PerCP-conjugated anti-CD8^+^ (clone 53-6.7) and FITC-conjugated anti-CD25 (clone 7d4), all from BD Biosciences. Regulatory T cells (Treg) were identified with the Mouse Regulatory Staining kit (eBiosciences). FACS^®^ Canto (BD Biosciences) was used for flow cytometry acquisition and analyses were performed with the FACS^®^ Diva software.

### Statistical analyses

Where indicated statistical analyzes were performed using GraphPad Prism software. Unpaired events were compared with a Mann-Whitney Test. Multiple comparisons were tested with a Kruskal–Wallis assay followed by Dunns post test to compare the different pairs. Survival curves were compared with a Log-rank test.
